# Transcription Factor DOF4.1 Regulates Seed Longevity in Arabidopsis *via* Seed Permeability and Modulation of Seed Storage Protein Accumulation

**DOI:** 10.3389/fpls.2022.915184

**Published:** 2022-07-01

**Authors:** Regina Niñoles, Carmen Maria Ruiz-Pastor, Paloma Arjona-Mudarra, Jose Casañ, Joan Renard, Eduardo Bueso, Ruben Mateos, Ramón Serrano, Jose Gadea

**Affiliations:** Instituto de Biología Molecular y Celular de Plantas, Universitat Politècnica de València-Consejo Superior de Investigaciones Científicas, Ciudad Politécnica de la Innovación, Valencia, Spain

**Keywords:** seed longevity, seed storage proteins, cruciferin, DOF4.1, transcription factor

## Abstract

Seed longevity is modulated by multiple genetic factors in *Arabidopsis thaliana*. A previous genome-wide association study using the Elevated Partial Pressure of Oxygen (EPPO) aging assay pinpointed a genetic locus associated with this trait. Reverse genetics identified the transcription factor DOF4.1 as a novel seed longevity factor. *dof4.1* loss-of-function plants generate seeds exhibiting higher germination after accelerated aging assays. DOF4.1 is expressed during seed development and RNAseq data show several putative factors that could contribute to the *dof4.1* seed longevity phenotype. *dof4.1* has reduced seed permeability and a higher levels of seed storage proteins mRNAs (cruciferins and napins) in developing seeds, as compared to wild-type seeds. It has been reported that mutant lines defective in cruciferins or napins present reduced seed longevity. The improved longevity of *dof4.1* is totally lost in the quadruple mutant *dof4.1 cra crb crc*, but not in a *dof4.1* line depleted of napins, suggesting a prominent role for cruciferins in this process. Moreover, a negative regulation of *DOF4.1* expression by the transcription factor DOF1.8 is suggested by co-inoculation assays in *Nicotiana benthamiana*. Indeed, *DOF1.8* expression anticorrelates with that of *DOF4.1* during seed development. In summary, modulation of DOF4.1 levels during seed development contributes to regulate seed longevity.

## Introduction

Seed longevity is an essential trait for plants with economic importance. Prolonged storage reduces seed viability, with many crops having short life spans when stored in an uncontrolled environment (Leprince et al., 2017). Seed longevity is a multifactorial trait, and an increasing number of genetic factors have been identified, mainly in model species, using reverse genetics, genome-wide association studies or coexpression network analysis (Righetti et al., 2015; Renard et al., 2021). A main strategy to circumvent seed deterioration is embryo protection from oxidative damage of cellular components such as lipids, nucleic acids and proteins (Bailly et al., 2008; Rajjou and Debeaujon, 2008; Rajjou et al., 2008), that would decrease seed germination rate or increase frequency of abnormal seedlings. At the interface between the embryo and the external environment, the seed coat is the first front to minimize oxidation from outside. In this regard, seed coat lipidic barriers such as cutin and suberin, that confer impermeability, have proven to be essential for seed longevity (Renard et al., 2020a,^2021^). Moreover, the ability of some types of seeds to transform the cytoplasm into a glassy state, which reduces metabolic activity and hence oxidation (named orthodox seeds), allow them to survive for decades in extreme dehydration (Buitink et al., 2000; Rajjou and Debeaujon, 2008). This glassy cytoplasm is proposed to be stabilized by oligosaccharides (i.e., raffinose, RFOs) and by the highly hydrophilic late embryogenesis abundant (LEA) proteins. LEA accumulation correlates with longevity acquisition (Chatelain et al., 2012) and decreases with aging (Rajjou et al., 2008), and down-regulation of the Arabidopsis *LEA14* gene affects seed longevity (Hundertmark et al., 2011). Small heat-shock-proteins sHSPs also play a role in seed longevity, likely preventing aggregation in the glassy-state and assisting in protein folding (Almoguera et al., 2012). Other protective mechanisms comprise passive antioxidant defenses, such as seed coat flavonoids (Debeaujon et al., 2000), tocopherols (Sattler et al., 2004), or glutathione (Kranner et al., 2006), as well as detoxifying enzymes, such as superoxide dismutases (Le et al., 2010; Lee et al., 2010) or the NADP-MALIC ENZYME 1, which reduces the levels of protein carbonylation during seed storage (Yazdanpanah et al., 2019).

Seed storage proteins (SSPs) accumulate significantly during seed maturation, and constitute the major source of reduced nitrogen for the growing seedlings. Seeds of *A. thaliana* contain two main classes of SSPs, cruciferins and napins. Cruciferins are 12S globulins synthesized as preproproteins, proteolytically cleaved into α and β subunits. Mature 12S globulins are 12-mers composed of six α and six β polypeptides (Adachi et al., 2003). Napins are 2S albumins that accumulate as heterodimers consisting also of two subunits generated by cleavage of a precursor (Krebbers et al., 1988). *Arabidopsis thaliana* contains four genes encoding cruciferins (*CRUCIFERIN A (CRA*, At5g44120*), CRUCIFERIN B (CRB*, At1g03880*), CRUCIFERIN C (CRC*, At4g28520), and *CRUCIFERIN D (CRD*, At1g03890) and five genes encoding napins, named as *SESA1* to *5* (van der Klei et al., 1993; Wan et al., 2007). Comparative proteomics of near isogenic lines (NILs) of Arabidopsis with introgressed genomic regions at seed longevity QTLs (Nguyen et al., 2012) suggested that SSPs are important for seed longevity, and knock-out mutants lacking CRA, CRB, and CRC or reduced levels of napins presented reduced seed longevity (Nguyen et al., 2015). Due to their abundance and their high affinity for oxidation, SSPs are potent ROS buffering system, protecting other proteins from oxidation. In fact, the level of carbonylated proteins after aging is increased in a triple *crua crub cruc* mutant (Nguyen et al., 2015). Therefore, understanding the dynamics of seed storage protein accumulation and their regulators reveals as a novel way to modulate seed longevity.

SSPs are synthesized during seed filling, increasing steadily from 10 to 17 days after pollination, and accounting for nearly 60% of the Arabidopsis dry seed protein content (Baud et al., 2002). The B3-domain-containing transcription factors FUSCA3 (FUS3), ABSCISIC ACID INSENSITIVE3 (ABI3) and LEAFY COTYLEDON2 (LEC2) are master regulators of seed development controlling most seed maturation processes, including seed storage proteins synthesis (Verma et al., 2022). SSP promoters contain elements with which B3-domain proteins interact (Bäumlein et al., 1986; Ezcurra et al., 2000; Reidt et al., 2000) and most of them are direct targets of FUS3, LEC2, and ABI3 (Braybrook et al., 2006; Mönke et al., 2012; Wang and Perry, 2013). Consequently, *abi3, lec2*, and *fus3* mutants exhibit reduction of SSP accumulation, and also reduced seed longevity (Parcy et al., 1997; Sugliani et al., 2009). LEAFY COTYLEDON1 (LEC1), another master regulator of seed development, activates the synthesis of SSPs *via* FUS3 and ABI3 (Kagaya et al., 2005). Gibberellins (GAs) negatively regulates the synthesis of SSPs. The DELLA protein RGL3 facilitates ABI3 transcriptional activation *via* protein–protein interaction, thus promoting the expression of SSP synthesis genes. Decrease of GAs and increase of abscisic acid during seed maturation (Yan and Chen, 2017) results in ABI3 and RGL3 accumulation and SSP synthesis.

SSPs expression arrest is also genetically regulated. At the end of the seed filling stage, *SSPs* genes are down-regulated and SSP accumulation ceases (Baud et al., 2002). Repression of the whole *LEC1* network is performed by members of the VP1/ABI3-LIKE (VAL) family of B3 domain transcription factors (Suzuki et al., 2007) or by the chromatin-remodeling factor PICKLE (Ogas et al., 1999). A more specific mechanism is the repression of specific seed maturation genes while allowing continued expression of others. One example is the French bean *ROM2* gene, a bZIP transcription factor expressed after storage protein expression. ROM2 binds to the phaseolin G-box promoter region and represses phaseolin expression (Chern et al., 1996). Seed storage protein synthesis is also linked to available nitrogen (Lhuillier-Sounde’le’ et al., 1999). During seed filling, seeds become sink tissues that rely on remobilization of nutrients, especially from senescent leaves, being nitrogen import into the seed a rate-limiting step (Havé et al., 2017). Several lines of evidence suggest the existence of sensors that monitor and adjust seed protein contents to nitrogen availability. For example, transgenic plants overexpressing storage proteins result in a compensatory reduction of the endogenous ones (Hagan et al., 2003; Scossa et al., 2008), and the TaNAM-B1 transcription factor present in ancestral wheat varieties accelerates leaf senescence and increases seed protein content (Uauy et al., 2006).

Recent genomics and systems approaches have revealed the intricate regulatory network controlling seed maturation (Holdsworth et al., 2008; Santos-Mendoza et al., 2008; Lee et al., 2010; Sreenivasulu and Wobus, 2013), that includes the expression of sets of transcriptions factor acting in different seed compartments at different stages of development, regulating many of the abovementioned events. DOF transcription factors are plant-specific transcription factors containing an N-terminal DOF (DNA-binding one finger) domain and a C-terminal region for transcriptional regulation. Arabidopsis contains 37 potential *DOF* genes (Yanagisawa, 2002; Yang et al., 2006; Moreno-Risueno et al., 2007; Le Hir and Bellini, 2013; Noguero et al., 2013), classified into seven groups discerning mainly in their C-terminal part, which confers them a broad range of biological functions in different plant-specific scenarios, such as photosynthetic carbon assimilation, light-regulated gene expression, accumulation of seed storage proteins, germination, dormancy, response to phytohormones and flowering time (Gupta et al., 2015). In the recent years, DOF proteins have been identified that bind to plant genes and interact with other transcription factors involved in seed development (reviewed in Ruta et al., 2020). Among them, the maize P-box-containing DOF transcription factor regulates SSP accumulation by binding to the prolamin box in zein gene promoters, interacting with the bZIP transcriptional activator Opaque2 (Vicente-Carbajosa et al., 1997), the maize ZmDOF36 protein positively controls starch accumulation (Wu et al., 2019), and the soybean *GmDof4* and *GmDof11* genes enhance lipid content in Arabidopsis seeds (Wang et al., 2007). DOF transcription factors are also involved in other aspects of seed development, such as suberin biosynthesis in the outer integument, which further influence seed longevity. In Arabidopsis, the *DOF1.5/COG1* gene is a negative regulator of light perception that controls the expression of the peroxidases PRX2 and PRX25, involved in the polymerization of suberin in the seed coat (Renard et al., 2020a). Also, the Arabidopsis DOF4.2 protein has been proposed to be involved in seed coat composition and mucilage production (Zou et al., 2013). Given the diversity in structure of the different DOF proteins, the discovery of more members of this family involved in seed development will be of great value to understand the molecular events controlling seed quality traits such as seed longevity.

In this study, we identified the transcription factor DOF4.1 as a negative regulator of seed longevity. *dof4-1* loss-of-function mutant exhibited enhanced seed viability after artificial aging assays. Transcriptomic analysis of *dof4-1* showed significant accumulation of transcripts of several longevity-related genes, especially SSPs. Seed permeability is reduced in *dof4-1* mutants, and lipid polyester layer is thicker in these mutants, suggesting also a role for DOF4.1 in seed coat lipid barriers development. The enhanced seed longevity of *dof4-1* is abolished in the quadruple mutant *dof4.1 crua crub cruc*, suggesting that increased cruciferin expression greatly contributes to the *dof4-1* phenotype. Moreover, a functional link is suggested between DOF4.1 and DOF1.8 transcription factors. DOF1.8 repressed *DOF4.1* expression *in Nicotiana* leaves and expression of both transcription factors anticorrelates during seed development. However, *dof1.8* mutants present similar longevity than wild type, suggesting the existence of additional cellular factor controlling *DOF4.1* expression.

## Materials and Methods

### Plant Material and Growth Conditions

*dof4.1-1* (Salk_076064), *dof4.1-3* (GABI_383E02), and *dof1.8-1* (Salk_130584) mutants were obtained from the Nottingham Arabidopsis Stock Centre (NASC). *cra crb crc (crabc)* mutant and RNAi napin line (transgenic line for napin silencing through RNAi), generated by Withana-Gamage et al. (2013), were a gift from Dr. Bentsink laboratory.

*Arabidopsis thaliana* seeds were surface sterilized with 70% ethanol 0.1% Triton X-100 for 15 min and rinsed three times with sterile water. Seeds were stratified for 3 days at 4^°^C. Germination was carried out on plates containing 25 ml Murashige and Skoog (MS) salts with 1% (w/v) sucrose, 10 mM 2-(N-morpholino) ethanesulfonic acid and 0.9% (w/v) agar, pH was adjusted to 5.7. To obtain fresh seeds, Arabidopsis plants were grown under greenhouse conditions (16 h light/8 h dark, at 23 ± 2^°^C and 70 ± 5% relative humidity) in pots containing a 1:2 vermiculite:soil mixture. Control and mutant plants were grown simultaneously.

### Artificial Aging Assays

Seeds used for testing were harvested at the same time and stored under the same conditions for at least 2 weeks prior to the experiment. A control germination assay without aging was used to discard genotypes with affected germination. The Elevated Partial Pressure of Oxygen (EPPO) (Groot et al., 2012), was performed with 5 bar O_2_ and 10% relative humidity of for 9 months. For the accelerated-aging treatment, imbibed seeds were aged at 40^°^C for 32–36 h. Natural seed aging treatment consisted of dry seed storage at room temperature (20–25^°^C, 40–60% RH) for 18 months. Except in accelerated aging, all treatments were performed prior to seed sterilization and stratification. After the treatment, seeds were sown on MS medium and seedling establishment with green cotyledons was scored after 9 days. All the assays were performed with three biological replicates using 50 seeds per replicate. *T*-tests were used to determine significant differences between genotypes.

### Biochemical Staining Assays

Triphenyltetrazolium salt penetration assay was performed as described by Molina et al. (2008). Briefly, seeds were incubated in the dark in 1% (w/v) tetrazolium red at 30^°^C for 2 and 4 days. Then, formazan was extracted and quantified as the absorbance at 485 nm. For mucilage staining, seeds were first imbibed in water during 3 min and then incubated in 0.2% Ruthenium Red solution for 15 m with agitation. After washing with water, photographs were taken with a Nikon’s Eclipse E600 microscope.

Seed coat suberin staining was performed on mature seeds with Sudan Red 7B as described by Bueso et al. (2016).

### Transcriptomic Assays and RNA Data Analysis

RNA was extracted from developing seeds (9 days after pollination, DAP), as described before (Oñate-Sánchez and Vicente-Carbajosa, 2008). Two replicates were performed for wild-type seeds, and three replicates for *dof4.1-3* seeds. Twenty-million paired-ends 50 nt reads per library (40 million in total) were sequenced. After adaptor removal and low-quality trimming of raw reads with cutadapt (Martin, 2011), clean reads were quality assessed with FastQC and mapped to the TAIR10 Arabidopsis thaliana genome using HISAT2 (Kim et al., 2015). Gene counts were then obtained with htseq-count (Anders et al., 2015) and used for differential expression analysis with DESeq2 (Love et al., 2014). Significative genes (p-adjusted (FDR) < 0.05 and expression in *dof4.1* at least two-fold higher or lower than in the wild type) were used for functional analysis using Panther (Mi et al., 2021). Redundant GO terms were removed using ReviGO (Supek et al., 2011) and remaining GO terms were visualized using bubble plots. Data have been submitted to GEO repository under the accession number GSE198206.

### qRT-PCR Expression Assays

RNA was extracted from developing seeds (3-21 DAP) as above. DNA removal was performed with DNAse kit (E.Z.N.A). About 500 ng RNA was reverse-transcribed using the Maxima first-strand cDNA synthesis kit (Thermo Fisher Scientific) according to the manufacturer’s instructions. qRT-PCR was performed with the PyroTaq EvaGreen qPCR Mix Plus (ROX; Cultek S.L.U., Spain) in a total volume of 20 μl using an Applied Biosystems 7500 Real-Time PCR System (Thermo Fisher Scientific). Data are the mean of three replicates. Relative mRNA abundance compared to AT5G55840, ASAR1 was calculated, using the comparative ΔCt method. Primers for qRT-PCR are listed in Supplementary Table 1.

### Constructs and *Agrobacterium* Transformation

To obtain pUBQ10:DOF4.1-HA construct, the cDNA of DOF4.1 was amplified from wild-type seeds cDNA, using the following primers, that include *Bam*HI restriction sites at both ends of the gene: DOF4.1 Bam FP: TAGCGGATCCATGGACCATCATCAGTATCAT and DOF4.1 Bam RP: TGAGGGATCCCCATGTTGGTCCACCACTAT. After amplification, it was cloned into the pCR8 vector. Then, the cDNA was introduced into pTEX:2xHA vector using *Bam*HI sites. Finally, the cDNA DOF4.1-HA fragment was obtained by digestion with *Sma*I and *Sal*I and ligated into the pUBQ10 vector.

To obtain *pDOF4.1:GFP* construct, the promoter of DOF4.1 (1,500 bp upstream the start codon) was amplified using the following primers (pDOF.FP: CCAAAACCAAACAGAATTGATTCCC and pDOF.RP: AGTAATTAATCCCTGTAATAAGTATACGTATG), subcloned into pCR8 vector, and finally into pMDC107 through GATEWAY technology.

*p35S:DOF1.8* was generated using the Goldenbraid (GB) technology (Sarrion-Perdigones et al., 2014). First, DOF1.8 cDNA sequence was domesticated and introduced in the pUPD2 vector following the instructions of GoldenBraid4.0 webpage. Primers for DOF1.8 domestication are listed in Supplementary Table 2. Then, a GoldenBraid multipartite assembly reaction was performed to join the generated DOF1.8 cDNA with the 35S promoter (GB0030) and a NOS terminator (GB0037) GB parts (in the pUPD2 vector) and to introduce the resulting transcriptional unit (TU) into the pDGB_α1 Goldenbraid destination vector. *E. coli* competent cells were transformed with the different constructs and proper cloning was proved by plasmid sequencing. The obtained constructs were introduced into *Agrobacterium tumefaciens* strain GV3101 for transient (in Nicotiana) or stable expression (in *dof4.1-3* mutant in Arabidopsis, using the floral dipping method).

### Transient Expression Assays in *Nicotiana benthamiana*

Transient expression assays were performed by agroinfiltration of 4-weeks-old *Nicotiana benthamiana* leaves. Agrobacterium cultures containing *pDOF4.1:GFP* and *p35S:DOF1.8* were grown until OD:0,2 and leaves were infiltrated with *pDOF4.1:GFP* with or without *p35S:DOF1.8*. *p35S:YFP* (GB0209) was employed as a positive control and *p35S:DOF1.8* alone was used as negative control. In all cases, *p35S:P19* silencing suppressor (GB0108) was coinfiltrated and *pDGB1alpha1_SF* (GB0106), a construct with a *Solanum lycopersicum* intergenic region (an “inert” DNA fragment), was used, when necessary, to equilibrate the final concentration of each Agrobacterium culture in the final mixes. 3 biological replicates were performed for positive and negative controls and 6 biological replicates for assaying DOF4.1 expression. After 5 days, GFP was observed in a confocal ZEISS-LSM710 microscope. Laser used was Argon with an excitation lambda of 488 nm, and an emission spectrum of 500–550 nm was registered.

## Results

### Loss-of-Function of the DOF4.1 Transcription Factor Increases Seed Longevity

**FIGURE 1:**
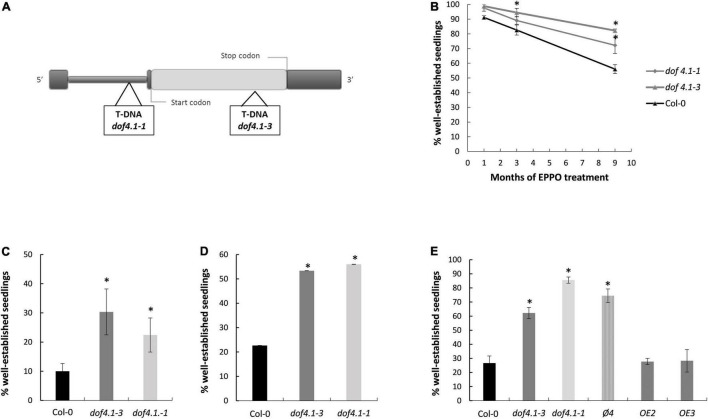
*dof4.1* seeds exhibit increased longevity. **(A)** Scheme of DOF4.1 locus showing the localization of T-DNA insertions in *dof4.1-1* and *dof4.1-3* alleles. **(B)** Elevated Partial Pressure of Oxygen (EPPO) treatment: Seeds were sown on MS plates after maintaining them during 1, 3, or 9 months at 5 bar O_2_ and 10% RH. **(C)** Accelerating Aging Treatment: seeds were imbibed in water and incubated at 40^°^C for 36 h. **(D)** Natural seed aging treatment: dry seeds were maintained at room temperature and ambient humidity (20–25^°^C, 40–60% RH) for 18 months and sown on MS plates. **(E)** Accelerated aging treatment in *dof4.1-3* lines overexpressing DOF4.1 under the control of pUBQ10 promoter. Seeds were imbibed in water and incubated at 40^°^C for 36 h. Percentage of established seedlings was recorded after 9 days growing on MS media. Bars represent the average and standard errors of three replicates with 50 seeds per line. Col-0, Columbia 0. φ4: Transgenic plant transformed with the empty pUBQ10 vector used to generate the overexpression lines. OE: overexpression lines. Asterisks indicate significant differences with WT (*P* < 0.05) between samples in two-tailed Student’s *t*-test.

Previously, a genome-wide association study on Arabidopsis had revealed a genomic region on chromosome 4 associated with seed aging after the Elevated Partial Pressure of Oxygen (EPPO) assay (Renard et al., 2020b). To identify the causal agent of this association, genes around the significant polymorphism (SNP) were selected as case studies for reverse genetic analysis. The associated SNP was in linkage disequilibrium with the AT4G00940 locus, encoding a Dof-type transcription factor (DOF4.1). The DOF4.1 protein belongs to the group III of DOF transcription factors of Arabidopsis, containing the binding domain in the N-terminal part, a characteristic C-terminal part shared by all members of this group, and a LPDLNP motif shared only by two DOF proteins of the group (DOF2.5/DAG2 and DOF3.7/DAG1), involved in seed germination (Gualberti et al., 2002; Yanagisawa, 2002). According to the Arabidopsis eFP Browser (Winter et al., 2007) DOF4.1 is expressed during seed development, making this gene a putative candidate for further analysis (Supplementary Figure 1). Two T-DNA alleles were selected for aging experiments. The *dof4.1-1* allele has the T-DNA inserted in the leader intron of this gene (Ahmad et al., 2013). RT-PCR experiments on homozygous plants show that this allele still transcribes some *DOF4.1* mRNA. However, this mRNA includes the T-DNA in the 5′-UTR (Supplementary Data Sheet 1), which will presumably interfere in ribosome scanning and translation of the DOF4.1 protein. The *dof4.1-3* allele contains a T-DNA in the second exon (Figure 1A). RT-PCR experiments show that this mutant is not able to transcribe a mRNA, and we consider it a knock-out allele (Supplementary Data Sheet 1).

Artificial aging assay (EPPO) over *dof4.1-1 and dof4.1-3* seeds indicate that DOF4.1 function is hampering seed longevity. After 9 months on high oxygen conditions, 72% of *dof4.1-1* and 83% of *dof4.1-3* seeds could germinate, against only 56% of wild type Col-0 seeds (Figure 1B). A similar response was obtained under a wet-aging artificial assay (accelerated aging assay, Figure 1C) and also after naturally aging the seeds for 18 months on ambient conditions in the laboratory (Figure 1D). In these assays, seeds of both *dof4.1* alleles are better storable than wild type, *dof4.1-3* seed presenting slightly higher longevity than *dof4.1-1* in EPPO and ambient assays. Complementation of the *dof4.1-3* mutant with a wild-type *DOF4.1* gene abolished the seed longevity resistance observed in the mutant, indicating that this phenotype is due to the lack of the DOF4.1 protein (Figure 1E). These results indicate that the presence in seeds of a functional DOF4.1 protein is detrimental for seed longevity under different storage conditions.

### Transcriptomic Experiments in *dof4.1-3* Seeds Suggest a Role for DOF4.1 in Seed Development

**FIGURE 2:**
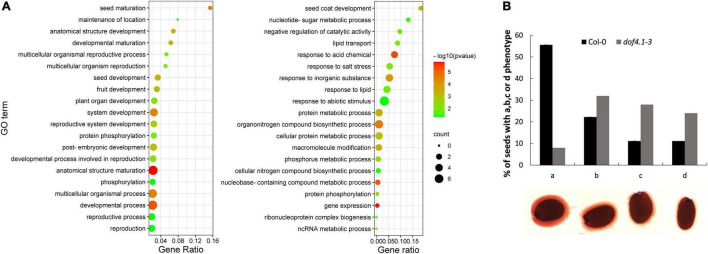
Seed development is affected in *dof4.1-3*. **(A)** Top-20 non-redundant enriched gene-ontology (GO) categories in *dof4.1-3* upregulated (left panel) and downregulated (right panel) genes. Biological Process enrichments were obtained using Panther and filtered using REVIGO to remove semantically redundant terms. Terms were pruned by REVIGO frequency (terms with frequency > 15% were removed) and ranked by dispensability. Gene ratio: Ratio of upregulated genes in a given category divided by total number of genes in this category. Counts: Number of upregulated genes in a given category. -log p (*p*-value in log scale after false discovery rate correction). **(B)** Histogram showing visual scoring of mucilage phenotypes, from perfect (a) to severely affected mucilage (d), after staining with ruthenium red in Col-0 and *dof4.1-3* seeds. 70 seeds were analyzed per genotype. Col-0, Columbia 0.

To gain insight into the mechanisms by which DOF4.1 is affecting seed longevity, the transcriptome of 10 days after fertilization (DAP) *dof4.1-3* seeds was compared to wild-type‘s. 259 and 623 genes were found up-regulated and down-regulated in *dof4.1-3*, respectively (Supplementary Table 2). The top 20 significant categories (gene ontology, biological process) among the two group of genes are shown in Figure 2A. Although their link to seed longevity is not straightforward at first glimpse, it is remarkable the enrichment of categories such as “seed maturation” (GO: 0010431) among the upregulated genes, and “seed coat development” (GO: 0010214), among the downregulated genes, suggesting a role for the *DOF4.1* gene in seed-related processes. A concerted upregulation was found among seed storage protein genes (cruciferins and napins), namely the ones encoding the CRA (3.0-fold), CRB (3.83-fold), CRC (3.6-fold) and CRD (5.7-fold) globulins, as well as in those encoding the SESA1 (AT4G27140) (5.7-fold), SESA2 (AT4G27150) (2.7-fold) and SESA5 (AT5G54740) (3.0-fold) 2S albumins. The *SESA4* gene (AT4G27170) was also upregulated (2.26-fold), but slightly below the criteria we used for significance (adjusted *p*-value 0.06). Moreover, the SSP-inductor *ABI3* gene was also up-regulated in the *dof4.1-3* mutant (1.85-fold). Surprisingly, the gene encoding the master regulator LEC1 was significantly repressed (2.65-fold), perhaps obeying to an SSP-dependent feedback mechanism. Moreover, also enriched on the upregulated genes was the category lipid droplet (GO: 0005811), specialized organelles acting as seed lipidic reservoirs. Other upregulated genes involved in seed maturation with a putative link with seed longevity are the *DELAY OF GERMINATION (DOG1)*, the *ABCG6* gene, encoding a suberin transporter and four late embryogenesis abundant (*LEA*) genes. Finally, we found upregulated genes involved in oxidative stress mitigation, such as the superoxide dismutase *MSD2*, the peroxiredoxin *PER1*, the orthologous gene of the rice *OsLpa1* gene, involved in phytic acid biosynthesis, or the *VTC5* gene, involved in the synthesis of ascorbic acid, as well as the *RF4* raffinose synthase and the NADP-Malic enzyme 1(*NADP-ME1*) genes. Any of them could potentially contribute to the extended longevity phenotype found in this mutant.

Among the *dof4.1-3* down-regulated genes included in the enriched “seed coat development” category, we found up to seven genes related to mucilage development, such as the *MYB61* and *KNAT7* transcription factors and the *MUM4, MUM5, MUCI21, MUCI10*, and *URGT2* genes. Moreover, the enriched category “negative regulation of catalytic activity” (GO: 0043086) includes ten downregulated pectin methylesterase inhibitor-related proteins (PMEI). Mucilage is mainly composed of pectin, and seed PMEIs regulate the degree of methylesterification of homogalacturonan, which affect mucilage properties. Among them, the *PMEI6* gene, specifically expressed in seed coat epidermal cells and necessary for proper mucilage development (Saez-Aguayo et al., 2013), was also downregulated in *dof4.1-3*. We then investigated the ability of *dof4.1* seeds to properly develop mucilage, using ruthenium red staining. When Arabidopsis wild-type seeds were hydrated, the seed coat mucilage swelled rapidly, and a dense pink-stained capsule was observed after staining. In contrast, *dof4.1* displayed a lighter staining and reduced thickness of the inner mucilage layer, suggesting a looser pectin network in this mutant (Figure 2B). These results suggest that DOF4.1 is involved in different aspects of embryo and seed coat development.

### Reduced Seed Permeability and Seed Storage Protein Accumulation in *dof4.1* Mutants Explain Their Longevity Phenotype

**FIGURE 3:**
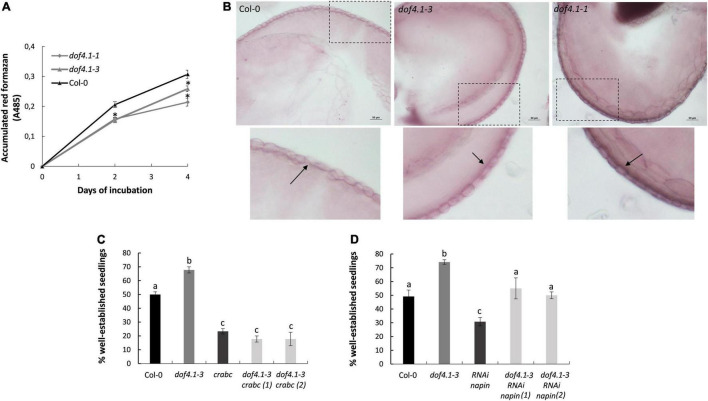
*dof4.1*mutants present reduced seed permeability and *dof4.1-3* increased SSPs content determine its higher longevity. **(A)** Seed permeability of *dof4.1* mutant. Quantitative time course of formazan accumulation in Col-0, *dof4.1-1*, and *dof4.1-3* seeds. Wild type (Col-0) and mutant seeds were incubated during 2 or 4 days in 1% tetrazolium at 30^°^C and then formazan was extracted and quantified. Data (absorbance at 485 nM) are the mean and standard error of three biological replicates. Asterisks indicate significant differences with WT (*P* < 0.05) between samples in two-tailed Student’s *t*-test. **(B)** Sudan Red 7B staining of the subepidermal layer of the seed coat in wild type (Col-0), *dof4.1-3* and *dof4.1-1* dry seeds. Scale bars correspond to 50 μM. Lower panels amplify the indicated area to show a detail of the staining. Arrows point to the suberin layer Accelerating Aging Treatment over a quadruple mutant *dof4.1-3 cra crb crc* **(C)** and a transgenic RNAi line for napins in *dof4.1-3* background **(D)**. Seeds were imbibed in water and incubated at 40^°^C for 32 h. Percentage of established seedlings was recorded after 9 days growing on MS media. Different letters indicate significant differences with WT (*P* < 0.05) between samples in two-tailed Student’s *t*-test. Col-0, Columbia 0. *crabc, cra crb crc.* RNAi napin: transgenic line silenced in napins by RNA interference. Data are the mean and standard error of three biological replicates.

Seed coat lipid barriers are important structure contributing to seed longevity, limiting the diffusion of oxidizing molecules between the embryo and the surrounding environment. Given the involvement of DOF4.1 in seed coat development, we quantified seed coat permeability of *dof4.1* mutants, measuring tetrazolium salt uptake using the triphenyltetrazolium reduction method (Molina et al., 2008). As shown in Figure 3A, both *dof4.1-1* and *dof4.1-3* alleles accumulated lower levels of red formazans compared with the wild-type control, suggesting that seed permeability is reduced in these mutants, which could be a mechanism for their extended seed longevity. Next, the ability of *dof4.1* mutants to synthesize a lipid polyester layer in the seed coat was investigated using biochemical assay. After staining seeds with Sudan red to visualize seed coat suberized cell walls, wild-type seeds showed the characteristic pink layer in the outer integument of the seed coat. This layer was clearly thicker in *dof4.1* mutants, suggesting that lipid polyester deposition in seeds is enhanced when DOF4.1 is not present (Figure 3B). We next evaluated the contribution of *dof4.1* SSP levels on seed longevity. The effect of these proteins on seed longevity was reported in napins and cruciferins depleted lines, which were sensitive to artificial aging (Nguyen et al., 2015). To ascertain whether the increased expression of cruciferins observed in the *dof4.1-3* mutant could contribute to its extended seed longevity, a quadruple mutant *dof4.1-3 cra crb crc* was generated. Similarly, to investigate the contribution of napins increased expression in *dof4.1*, we crossed *dof4.1-3* with an RNAi line depleted in napins (Withana-Gamage et al., 2013). Accelerate aging assays show that the positive effect on longevity of the *dof4.1-3* mutant is abolished when all cruciferins are depleted (Figure 3C), indicating that a major contribution to longevity of the *dof4.1* mutants rely on the increased expression of cruciferins and that other putative contributing factors cannot compensate for the total depletion of these proteins. In the napins-depleted *dof4.1* mutant, the *dof4.1* background still can confer an advantage in seed longevity (Figure 3D), likely by the increased expression of cruciferins of this mutant, or by additional factors. In summary, these experiments indicate that loss-of-function of *DOF4.1* affects positively to seed impermeability. It also promotes napins and cruciferins accumulation. Cruciferin overaccumulation is determinant for the extended seed longevity observed in *dof4.1* mutants, although additional SSP-independent contributions are not discarded.

### DOF4.1 as a Modulator of Seed Storage Proteins Expression During Seed Development

**FIGURE 4:**
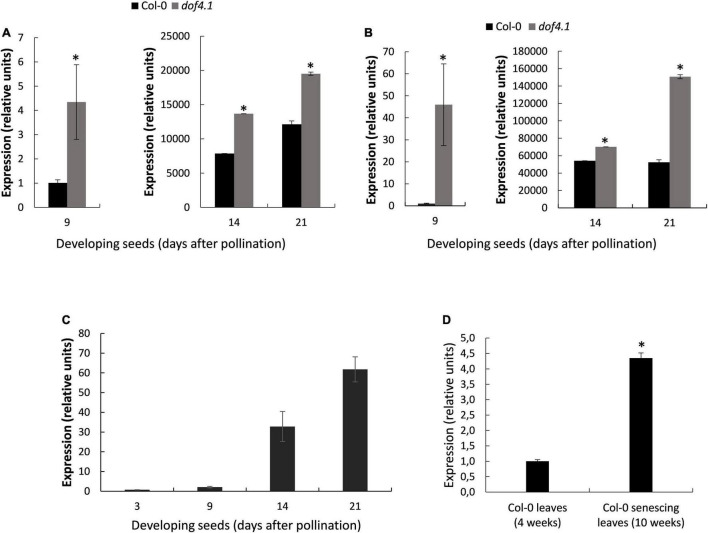
SSPs expression levels during seed development and *dof4.1* expression in developing seeds and senescing leaves. Expression levels of cruciferins **(A)** and napins **(B)** were determined by real-time quantitative PCR (qRT-PCR) in developing seeds (9, 14, and 21 DAP) of Col-0 and *dof4.1-3*. **(C)** Expression level of DOF4.1 was determined by qRT-PCR in developing seeds (3, 9, 14, and 21 DAP) and in senescing leaves (10 weeks after transferring to soil) compared to non-senescing leaves (4 weeks after transferring to soil) **(D)**. Data are the mean of three replicates. Significantly differing from Col-0 at *P* < 0.05 (*) using Student’s *t*-test. Col-0 = Columbia 0. DAP, Days After Pollination.

To understand the role of DOF4.1 in the seed, we first monitored the effect of its loss-of-function on SSP accumulation along seed development. Primers for RT-PCRs were designed for conserved regions of all four cruciferins, or for conserved regions of napins SESA1, 2 and 5 (see Supplementary Methods), to amplify cruciferins or napins in a single reaction. As shown in Figures 4A,B, expression of napins and cruciferins at 9 DAP followed the same profile as our RNA-seq results, with higher expression of the genes encoding both types of SSPs in *dof4.1-3* seeds as compared to wild type. This difference in expression is maintained or even increased at 14 and 21 DAP for both types of SSPs genes. We next studied the expression of *DOF4.1* along seed development. As shown in Figure 4C, *DOF4.1* start being expressed after 9 DAP, being progressively accumulated at 14 and 21 DAP. This indicates that expression of this transcription factor is triggered at later stages of seed development, when storage reserve accumulation in the embryo is reaching to an end and the seed is reaching its mature stage, suggesting that DOF4.1 is involved in reducing the expression of *SSP* genes at later stages of development.

Nitrogen availability is a rate-limiting step for SSPs synthesis. Leaf senescence is another process induced by nitrogen deficiency (Balazadeh et al., 2014). In order to ascertain whether *DOF4.1* could be also expressed in this nitrogen-dependent scenario, we compared expression of this gene in green well-expanded leaves vs. senescing leaves. As shown in Figure 4D, *DOF4.1* expression is 4.35-fold higher in senescing leaves, suggesting that this transcription factor could be involved in nitrogen sensing processes in different organs of the plant.

### *DOF4.1* Expression Is Repressed by DOF1.8

**FIGURE 5:**
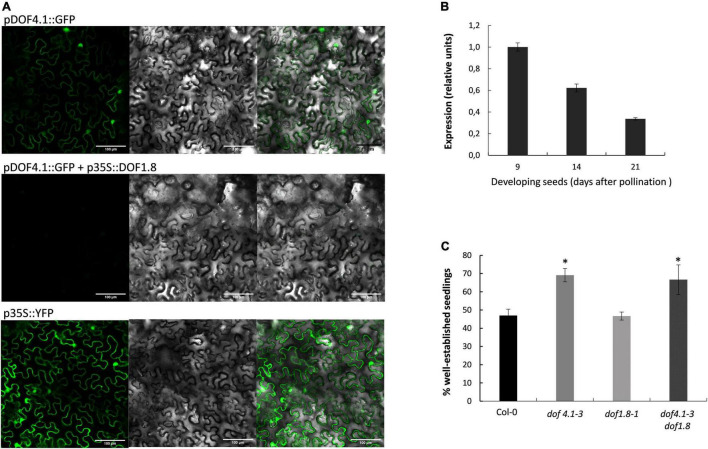
DOF1.8 transcription factor represses DOF4.1. **(A)** Representative images showing the transient expression of pDOF4.1:GFP in *Nicotiana benthamiana* leaves 5 days after agroinfiltration in the absence (upper panel) or in the presence (middle panel) of *p35S:DOF1.8*. The lower panel shows the expression of the positive control p35S: YFP. Scale bars 100 μM. **(B)** Expression level of DOF1.8 was determined by qRT-PCR in developing seeds (9, 14, and 21 DAP). DAP, days after polinization. **(C)** Accelerated aging assay of *dof1.8-1* mutant and *dof4.1-3 dof1.8-1* double mutant. Seeds were imbibed in water and incubated at 40^°^C during 32 h. The percentage of established seedlings (with green cotyledons) was recorded after 9 days growing on MS media. Data are the mean and standard error of three biological replicates. Asterisks indicate significant differences with wild type (*P* < 0.05) in two-tailed Student’s *t*-test. Col-0, Columbia 0.

DOF1.8 was identified as a putative regulator of *DOF4.1* expression in a high-throughput one-hybrid experiment (Brady et al., 2011), and transient expression analysis in Arabidopsis seedlings using a GAL4-binding assay suggested that DOF1.8 has transcriptional repressor activity (Ramachandran et al., 2020). To ascertain whether DOF1.8 could be acting as a transcriptional repressor of *DOF4.1*, a fusion plasmid containing the green-fluorescent protein (GFP) under the control of *DOF4.1* promoter (1.5 kb upstream of translational start) (*pDOF4.1:GFP*) was constructed and co-infiltrated into *N. benthamiana* epidermal cells together with a *p35S:DOF1.8* construct. At 5 dpi, GFP fluorescence was observed in the nuclei of plants infiltrated with *pDOF4.1-GFP* alone, indicating that the *DOF4.1* promoter is active in *Nicotiana* leaves. This signal was clearly reduced when this constructs was co-infiltrated with *p35S:DOF1.8*, suggesting that DOF1.8 repressed *DOF4.1* expression (Figure 5A). We next studied the expression of *DOF1.8* during seed development. As shown in Figure 5B, *DOF1.8* is expressed at 9 DAP, being progressively repressed at 14 and 21 DAP, consistent with the increased expression of *DOF4.1* in these time points. This anticorrelated expression support the hypothesis that DOF1.8 could be repressing *DOF4.1* transcription in seeds, although whether DOF1.8 is directly binding to the *DOF4.1* promoter or the repression is mediated by an intermediate factor is not known. To investigate whether DOF1.8 could also be involved in seed longevity *via* DOF4.1 regulation, we conducted aging assay on a loss-of-function *dof1.8* allele containing a T-DNA in the second exon (named *dof1.8-1*). Accelerated aging assays show that *dof1.8-1* seeds present similar longevity than wild type, whereas *dof1.8-1 dof4.1-3* double mutant maintains the reduced longevity observed for *dof4.1-3* (Figure 5C). This suggest the existence of redundant cellular factors that can substitute DOF1.8 to repress *DOF4.1* in seeds in a *dof1.8* background.

## Discussion

The three compartments of developing seeds (embryo, endosperm and seed coat) undergo rapid reprogramming events from fertilization until maturation in a complex regulatory network, comprising the activation by transcription factors of a precise set of target genes involved in multiple cellular processes (Gutierrez et al., 2007; North et al., 2010). Seed longevity, acquired at late stages of seed maturation, is a multifactorial trait that is regulated at different levels in these three compartments. Therefore, transcription factors involved in different aspects of seed development are likely to modulate seed longevity *via* their impact on downstream responses contributing to this trait. Mutants of the master regulators of seed development FUS3, LEC1, LEC2, and ABI3 are badly storable, likely by their inability to activate seed storage proteins, raffinose oligosaccharides (RFOs), and by their lack of chlorophyll degradation (Sugliani et al., 2009). APETALA2 is also involved in more than one compartment, and *ap2* mutants have reduced seed longevity, probably by multiple effects on the seed (Leon-Kloosterziel et al., 1994; Ohto et al., 2009). In other cases, the precise cause affecting longevity is more defined, as for example for the homeobox HB25, mediating apoplastic lipid barriers (Renard et al., 2021) for the flavonoid biosynthesis regulators, TT2, TT8, or TT16 (Debeaujon et al., 2000) or for the HEAT SHOCK FACTOR HSF9, activating small heat-shock-proteins (Prieto-Dapena et al., 2006; Tejedor-Cano et al., 2010). More transcription factors impacting seed longevity have been described, although their role is still uncertain. For example, WRKY3 and NFXL1 were identified a coexpression network analysis (Righetti et al., 2015), and KNAT7, SEPALLATE 3 or MYB47 in a genome-wide association study (Renard et al., 2020b). Loss-of-function mutants of these genes all exhibit reduced seed longevity. Here, we introduce a new player in the intricate regulatory network governing seed development, and suggest possible causes for the enhanced seed longevity of their loss-of-function mutant. The better storability of *dof4.1* seeds suggest that DOF4.1 acts negatively on processes that foster seed longevity. Analysis of the transcriptome suggest that this could be happening at least *via* the seed coat and the embryo. The reduced permeability of *dof4.1* mutants and the thickness of their lipid layers suggest that DOF4.1 could be negatively regulating seed coat lipid barriers, although whether this factor is regulating lipid levels or composition is not known. Increased expression of *DOG1* or *NADP-ME1* could be additional components contributing for the increased storability of *dof4.1* seeds. DOG1 is involved in multiple aspects of seed maturation and longevity, including the increase of compounds of the RFO pathway or accumulation of LEA and heat-shock proteins (Dekkers et al., 2016) and *dog1* mutants exhibits reduced seed longevity (Bentsink et al., 2006). Seeds of the *NADP-ME1* loss-of-function mutant have reduced seed viability and display higher levels of protein carbonylation than those of the wild type. NADP-ME1 catalyzes the oxidative decarboxylation of malate to pyruvate producing NADPH, and is proposed to protect seeds against oxidation (Yazdanpanah et al., 2019). Likely, all these factors contribute to the extended longevity found in *dof4.1* seeds. However, a major contribution for the *dof4.1* phenotype seems to be the increased expression of cruciferins. The importance of these reserves for longevity is demonstrated in the quadruple mutant *dof4.1 cra crb crc*, which completely abolished the enhanced longevity of *dof4.1.* The same effect was not observed in a *dof4.1* mutant depleted in napins, confirming that cruciferins, and not napins, are a major target for carbonylation, acting as buffers during storage and protecting other proteins from oxidation (Nguyen et al., 2015). This is consistent with the phenotype observed for the *transparent testa glabra 1 (ttg1)* mutant, which present reduced seed longevity (Debeaujon et al., 2000) despite accumulating more napins than wild type (Chen et al., 2015). The low longevity of this mutant is probably related to the function of TTG1 in seed coat development and flavonoid biosynthesis. DOF4.1, as well as TTG1 (Gonzalez et al., 2009), are positively regulating mucilage development; however, this structure is apparently not involved in seed longevity (Renard et al., 2021), and will not be discussed further in this study.

Dof transcription factors contain the characteristic one-finger domain (a multifunctional domain involved not only in DNA binding, but also in interactions with other proteins and in cell-to-cell trafficking), and a potential transcriptional activation or repression domain, normally in the C-terminal part of the protein (Yanagisawa, 2016). The predicted domain structure of DOF4.1 (Yanagisawa, 2002; Chen et al., 2013) do not allow to conclude whether this transcription factor is an activator or a repressor, which will require additional molecular studies. Negative regulation of longevity responses mediated by DOF4.1 could be achieved by direct repression of target genes, by activation of downstream negative regulators, or by interaction with signaling proteins. This last mechanism could be the case for MPK10, a mitogen-activated protein kinase involved in endosperm development and seed size (Xi et al., 2021) which interacts with WRKY10 and inhibits the expression of its target genes. Indeed, DOF4.1 interacts with MPK10 (Popescu et al., 2009), perhaps mediating these downstream responses in the endosperm.

This study positions DOF4.1 as an important player mediating the feedback regulation of the regulatory network governing accumulation of seed storage proteins. *SSPs* genes are directly regulated by the LEC2/FUS3/ABI3 and LEC1 master regulators of seed development, increasing their expression progressively during seed maturation. FUS3 suppresses *TTG1* expression, which inhibits the expression of 2S precursors. *TTG1* expression increases during late maturation, consistently with FUS3 decrease, and consequently, *SSP* expression also decrease. Similar to the profile of SSP accumulation observed here for *dof4.1, ttg1* mutants also present enhanced accumulation of SSPs (napins, but not cruciferins) along seed development (Chen et al., 2015). *DOF4.1* transcript levels also increase progressively along seed development, contributing to the inhibition of SSPs expression that take place at this late stages of seed development (napins and cruciferins). DOF4.1, however, is not a direct target of FUS3 (Wang and Perry, 2013), ABI3 (Mönke et al., 2012; Tian et al., 2020), nor LEC1 (Pelletier et al., 2017), although *DOF4.1* expression is repressed in *fus3* and *lec1* loss-of-function mutants (Yamamoto et al., 2014), which could be a new FUS3-dependent mechanism aimed to avoid excessive accumulation of SSPs at later stages of seed development. Interestingly, *DOF4.1* is highly expressed in senescing leaves. The nitrogen content of a leaf reaches the maximum level at completion of leaf expansion, and gradually decreases in the course of leaf senescence (Mae, 2004). Storage protein accumulation in seeds depends strongly on nitrogen availability (Lhuillier-Sounde’le’ et al., 1999; Miranda et al., 2001; Rolletschek et al., 2005), explaining the reserve compensation effect observed in biotechnological approaches aimed to increase the amount of seed proteins (Hagan et al., 2003; Scossa et al., 2008). It is tempting to speculate that seed nitrogen depletion at later stages of seed development could also be a signal activating DOF4.1, which would inhibit *SSP* expression independent of the FUS3 network. Interestingly, in a high-throughput interactome of nitrogen-associated metabolism, DOF4.1 was shown to bind the promoter of the *GLUTAMATE DEHYDROGENASE 2 (GDH2)* gene (Gaudinier et al., 2018), encoding a key enzyme involved in the incorporation of ammonia into organic compounds when remobilization of nutrients is required (Skopelitis et al., 2006), reinforcing the hypothesis of DOF4.1 as a nitrogen sensor.

DOF1.8 is able to repress *DOF4.1* expression (although this repression may be direct or mediated by an intermediate factor), and the anticorrelated expression of both genes in seeds suggest that this interaction could be biologically relevant *in vivo*. Where in the seed this interaction might be taking place is speculative and further studied will be needed, but the pattern of expression of these genes can envisaged some possibilities. *DOF1.8* expression is mainly restricted to vascular tissues (Ramachandran et al., 2020). The unique maternal vascular bundle in the Arabidopsis seed terminates at the end of the funiculus and releases its content into the testa (Robinson-Beers et al., 1992), suggesting that the molecular repression of *DOF4.1* by DOF1.8 in the seed coat could be taking place in this region. The symplastic connectivity within seed coat integuments (Stadler et al., 2005) would allow DOF4.1 to move to distal cells and regulate downstream processes in the outer integument, such as mucilage or lipidic barriers development, where DOF4.1 seems to be acting. Selective trafficking of transcription factors is widespread in plants (Han et al., 2014; Kitagawa and Jackson, 2017) and an intercellular trafficking motif has been reported as necessary for targeting proteins to plasmodesmata for cell-to-cell movement. Based on this, up to eight DOF transcription factors have been potentially considered as mobile (Lee and Zhou, 2012) and DOF4.1 is the canonical protein where this intercellular trafficking motif was discovered, allowing it to move between cells in roots and shoots tissues, and therefore opening the possibility to travel between cells through plasmodesmata also in seeds (Lee et al., 2006; Chen et al., 2013). A similar scenario could be hypothesized for the embryo up to globular stage, where a unique symplastic region exist, or for more developed embryos, with symplastic connectivity remaining between groups of cells belonging to similar tissues (Stadler et al., 2005).

Unfortunately, we could not observe a clear seed longevity phenotype in a *dof1.8* background. This could be explained by another DOF factor substituting DOF1.8 when this is not present. In fact, both DOF1.8 and DOF4.6, the most similar DOF factor to DOF1.8 in Arabidopsis, present a similar pattern of expression (Ramachandran et al., 2020). Similar examples have been described for other proteins of the DOF family. For example, mucilage content and composition are altered in transgenic plants overexpressing the DOF4.2 transcription factor, but mucilage development is normal in *dof4.2* (Zou et al., 2013). The redundancy in the DOF family, and the strong similarity among Dof DNA-binding domains suggests that Dof proteins display similar DNA-binding specificity, making plausible this hypothesis between members of the same sub-group of DOF proteins (Gupta et al., 2015; Yanagisawa, 2002). Alternatively, compensatory effects could maintain normal DOF4.1 levels in the cell, even in a mutant background.

This work represents a step forward in the knowledge of the complex transcriptional network regulating seed coat development and seed storage protein accumulation, identifies DOF4.1 as a novel player contributing to seed longevity and opens a door to further studying the role of its negative regulator, DOF1.8, in seed deterioration.

## Data Availability Statement

The datasets presented in this study can be found in online repositories. The names of the repository/repositories and accession number(s) can be found below: https://www.ncbi.nlm.nih.gov/geo/, GSE198206.

## Author Contributions

JG and RN conceived to the original idea, designed the experiments, and wrote the manuscript. RN, PA-M, JR, RM, JC, and CR-P performed the experiments. RN, EB, RS, and JG discussed the results and redesigned the experiments. All authors contributed to the article and approved the submitted version.

## Conflict of Interest

The authors declare that the research was conducted in the absence of any commercial or financial relationships that could be construed as a potential conflict of interest.

## Publisher’s Note

All claims expressed in this article are solely those of the authors and do not necessarily represent those of their affiliated organizations, or those of the publisher, the editors and the reviewers. Any product that may be evaluated in this article, or claim that may be made by its manufacturer, is not guaranteed or endorsed by the publisher.

## Supplementary Material

Supplementary Figure 1 Output image from the eFP (electronic fluorescent pictographic) browser showing the expression pattern of *DOF4.1* in the Developmental series generated by Schmid et al. (2005, Map of Arabidopsis Development) and colleagues from Detlef Weigel’s group in Tübingen. Each pictograph is colored according to the level of expression for the gene on of interest (Absolute) (http://bar.utoronto.ca/efp2/Arabidopsis/Arabidopsis_eFPBrowser2.html).

Supplementary Table 1 List of primers used in this study.

Supplementary Table 2 Lists of differentially-expressed genes in seeds at 10 DAP between *dof4.1-3* and wild type. Genes more expressed in *dof4.1-3* (sheet1) or in wild type (sheet 2) seeds. p-adj: adjusted *p*-value using false discovery rate correction (FDR).
