# Supplementary material for: Transcription Factor DOF4.1 Regulates Seed Longevity in Arabidopsis via Seed Permeability and Modulation of Seed Storage Protein Accumulation
Source: Front Plant Sci. 2022 Jul 1;13:915184. doi: 10.3389/fpls.2022.915184 (PMC9284063; doi:10.3389/fpls.2022.915184)
Supplement: Supplementary file 1 [file Data_Sheet_1.docx]

**Semiquantitative RT-PCR to study *DOF4.1* expression in *dof4.1-1* and *dof4.1-3* mutants**

To study the expression of DOF4.1 in *dof4.1* mutants, we performed semiquantitative RT-PCR using the primers listed below and represented in Figures 1A and 2A:

| F1 | 5’ AGAGTATGATCATGAGCACC 3’ |
| --- | --- |
| F2 | 5’ ATCTCTTTGAACATCAGGGATT 3’ |
| R1 | 5’ AGGATGATTCCGCTCCATAG 3’ |
| R2 | 5’ TTGCTTGAGTTGCATCTTGG 3’ |
| R3 | 5’ GGCTCCATTAGACAAAAGACAAAG 3’ |
| LBb1.3 | 5’ ATTTTGCCGATTTCGGAAC 3’ |
| AT5G55840 for | 5’ GATGATATTGCAGTTTGTCACCGT 3’ |
| AT5G55840 rev | 5’ CACTGTCTTGCTTGTCTTGTTCTG 3’ |

As shown in Figure 1B, using primers annealing downstream of the T-DNA insertion (F1 and R1) a band of 228 bp is obtained, both in wild type and in the *dof4.1-1* mutant, indicating that the gene is still transcribed in the mutant line. Band intensity indicates, however, that transcription is much lower in the mutant . Using F2 (whose sequence is interrupted by the leader intron) and R1 primers, a band of 884 bp is obtained in the wild type, but not in the mutant (Figure 1B), indicating that the intron is not spliced in the *dof4.1-1* mRNA*.* Confirming this, LBb1.3 (localized in the T-DNA) and R2 primers amplify a band of around 600 bp only in the mutant. Taken together, these results indicate that an aberrant transcript of *DOF4.1* is expressed in *dof4.1* mutant, that doesn’t splice the intron and includes the T-DNA. This aberrant *dof4.1-1* transcript is expressed at lower levels than a wild-type transcript in a wilt-type seed.


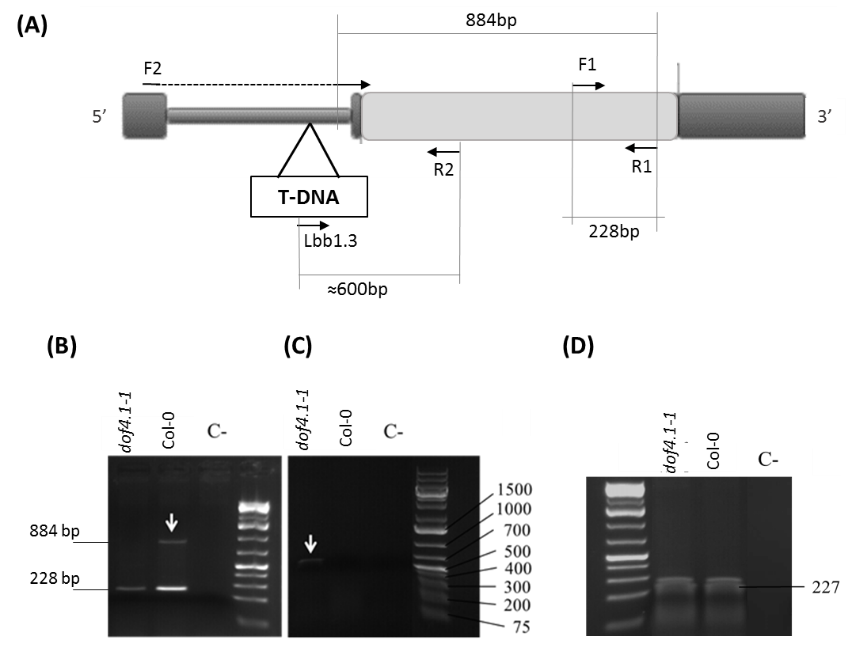


Figure 1: Semiquantitative RT-PCR to study *DOF4.1* expression in *dof4.1-1* mutant. (A) Scheme of the *DOF4.1* locus showing the localization of the T-DNA insertion and the primers employed in the amplifications. Amplified products in *dof4.1-1* and Col-0 backgrounds using F1, F2 and R1 primers (B), LBb1.3 and R2 primer (C) or primers amplifying a constitutive gene (AT5G55840) as a control. C-: PCR negative control.

For the *dof4.1-3* mutant, using primers localized upstream of the T-DNA insertion (F2 and R1, F2 sequence interrupted by the leader intron) a band of 278 bp is obtained (Figure 2B) only in the wild type, suggesting that *dof4.1-3* is a knock-out mutant.


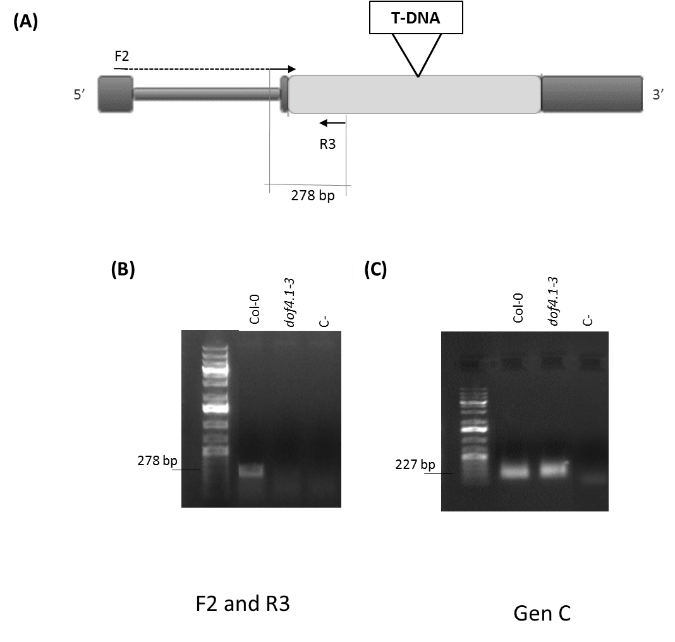


Figure 2: Semiquantitative RT-PCR to study *DOF4.1* expression in *dof4.1-3* mutant. (A) Scheme of the *DOF4.1* locus showing the localization of the T-DNA insertion and the primers employed for the amplification. Amplified products in Col-0 and *dof4.1-3* backgrounds using F2 and R3 primer (B) or primers amplifying a constitutive gene (AT5G55840) as a control (C). C-: PCR negative control.

**Seed storage proteins (SSPs) qRT-PCR primer design**

For *SSPs* RT-PCR, a multiple sequence alignment (clustal) was performed for all four cruciferins and conserved regions were selected for primer design, as shown below (blue boxes):

CRC ATGGTTAAGCTCAGCAATCTCCTCGTT--------GCAACCTTCGGGGTTCTCCTCGTCC

CRD ATGCATAAGCTTTTG--TTTTCTCTTCTCTCCGTCGTCTCACTCTCATTTCTCCTCTTCT

CRB -----ATGGGTCGAGTCTCATCTATTATCTCTTTCTCT---TTGACACTCTTGATCCTCT

CRA1 -----ATGGCTCGAGTCTCTTCTCTTCTTTCTTTCTGC---TTAACACTTTTGATCCTTT

::.* * * : **. * * . * * .** *

CRC TTAACGGCTGCCTTGCGAGGCAGTCACTTGGGGTTCCTCCTCAGCTACAGAACGAGTGTA

CRD TCCATGGCGCCGAGGCACGCCAGCG------AGA---GGCGCCGTTTCCAAACGCCTGCC

CRB TCAATGGCTACACTGCCCAACAG---------------------TGGCCCAACGAGTGCC

CRA1 TCCATGGCTACGCGGCTCAACAGGGTCAGCAGGG---TCAGCAGTTTCCGAACGAGTGCC

* .* *** * ** .. *** *. ****. ** .

CRC ACCTCGACAACCTAGATGTTCTCCAAGCCACCGAAACTATCAAGAGTGAAGCCGGTCAGA

CRD ATTTCAGCCAAATCAACAGCCTCGCGCCCGCTCAGGCGACGAAGTTCGAAGCCGGTCAGA

CRB AGCTCGATCAACTCAATGCGCTCGAACCATCCCAAATCATCAAGAGCGAGGGTGGTCGCA

CRA1 AGCTCGACCAGCTCAATGCGCTCGAGCCGTCACACGTACTGAAGAGCGAGGCTGGTCGCA

* **.. .* .*..* . *** .. * * * . . ***: **.* ****. *

CRC TCGAGTACTGGGACCACAACCACCCTCAGCTCCGATGTGTTGGTGTTTCCGTTGCTCGTT

CRD TGGAAGTATGGGACCACATGAGCCCTGAGCTCCGATGCGCCGGTGTAACGGTGGCTCGCA

CRB TCGAGGTCTGGGACCACCATGCACCCCAGCTCCGTTGCTCCGGCTTTGCCTTTGAGCGTT

CRA1 TCGAGGTGTGGGACCACCACGCTCCTCAGCTCCGTTGCTCAGGTGTCTCCTTTGCACGTT

* **. : *********.: ** *******:** ** * * * *. ** :

CRC ATGTAATTGAACAAGGCGGTCTTTACTTGCCCACCTTCTTCACTTCCCCAAAAATTTCCT

CRD TCACCCTTCAGCCCAATTCCATTTTCTTGCCCGCTTTCTTTAGCCCACCTGCCCTTGCTT

CRB TCGTCATTGAGCCTCAGGGTCTTTTCTTGCCCACTTTCTTGAACGCCGGCAAACTCACGT

CRA1 ACATCATCGAGTCTAAGGGTCTCTACTTGCCCTCTTTCTTTAACACCGCGAAGCTCTCTT

: . ..* *. . . .*.*:******* *.***** * *. .. .* * *

CRC ACGTCGTTCAAGGAACGGGTATCAGCGGAAGAGTGGTCCCTGGATGTGCCGAGACCTTCA

CRD ACGTTGTCCAAGGAGAAGGAGTTATGGGGACGATTGCTTCTGGTTGTCCTGAGACTTTTG

CRB TTGTTGTTCACGGAAGGGGTCTAATGGGAAGAGTTATTCCGGGATGCGCCGAGACGTTCA

CRA1 TCGTGGCTAAGGGACGAGGTCTTATGGGAAAAGTGATCCCTGGATGCGCCGAAACATTCC

: ** * .* *** .**: * * **.* ..* . * **:** * **.** **

CRC TGGACTCGCAGCCGATGCAAGGACAACAACAAGGCCAACCATGGCAAGGACGACAGGGAC

CRD CAGAAG--------TTGAAGGATC------------------------------------

CRB TGG-----------------AGTC------------------------------------

CRA1 AAG-----------------ACTC------------------------------------

.* . :*

CRC AACAAGGCCAACCATGGGAAGGACAGGGACAACAGGGACAACAAGGAAGACAAGGCCAAC

CRD ------------------------------ATCAGGAA----------G-----------

CRB ------------------------------ACCGGTAT----------T------TGGAG

CRA1 ------------------------------ATCAGAGT----------T------CCAAC

* *.* .:

CRC CATGGGAAGGACAGGGACAACAGGGACAACAAGGACGACAGGGACAACAAGGCCAACCAT

CRD ----------------------------------------------------------AG

CRB AA---------------------------------------------------------G

CRA1 CA------------------------------------------------------CGCT

CRC GGGAAGGACAGGGACAGCAGGGACAACAAGGGTTCCGTGACATGCACCAGAAGGTGGAAC

CRD GAGGAGGAGGAGACCC---G---GGTCGACGTTTTGAGGACATGCACCAGAAGTTGGAGA

CRB GTCAAGGTCAGGGTCA---G---AGTCAAGGGTTCCGTGACATGCACCAGAAAGTAGAGC

CRA1 TCGAAGGTCAAGGTCA---A---AGCCAGAGGTTCCGTGACATGCACCAGAAAGTGGAGC

.***: ..*. *. . . *.. * ** . **************. *.**..

CRC ATGTGAGACGCGGAGACGTCTTTGCCAACACTCCAGGCTCTGCCCACTGGATCTACAACT

CRD ATTTCCGGCGAGGGGATGTGTTTGCTTCGCTTGCCGGAGTTTCACAGTGGTGGTACAACC

CRB ACCTACGGTGCGGTGACACCATTGCAACACCATCTGGTGTAGCTCAATGGTTCTACAACA

CRA1 ACATTAGGAGCGGTGATACCATTGCCACAACACCCGGTGTAGCACAGTGGTTCTACAACG

* * .*. *.** ** . :**** :. . : * ** : * ** ***: ******

CRC CAGGAGAACAGCCACTTGTCATCATCGCTCTTCTCGACATCGCCAACTACCAAAACCAAC

CRD GCGGTGATTCCGATGCCGTCATTGTCATTGTTCTTGATGTCACCAACAGAGAAAACCAGC

CRB ATGGAAATGAGCCTCTCATTCTTGTTGCAGCCGCGGATCTCGCCAGCAACCAGAACCAGC

CRA1 ACGGACAGGAACCACTTGTCATCGTCAGCGTCTTCGATCTAGCCAGTCACCAGAACCAGC

**: * . .: .* .* .* . ** *..***. .. *.*****.*

CRC TCGACCGCAACCCTAGAGTGTTCCATTTGGCCGGAAACAACCAGCAGGGAGGCTT-TGGC

CRD TTGACCAAGTCCCTAGGATGTTCCAACTAGCCGGGAGCAGAACGCAAGAAGAAGAACAAC

CRB TTGACCGCAACCTTAGACCATTTTTGATAGCCGGAAACAACCCACAAGGGCAGGAATGGC

CRA1 TTGACCGCAACCCAAGGCCATTTTACTTAGCCGGAAACAACCCACAAGGTCAAGTATGGC

* ****...:** :**. .** : *.*****.*.**.....**.*. . : ..*

CRC GG--TTCACAGCAACAACAAGAACAGAAAAACTTGTGGAGCGGGTTCGACGCACAGGTCA

CRD --CATTAACGTGGCCATCAGG----CAACAACGCTTTCAGCGGTTTCGACCCAAACATAA

CRB TACAAGGCCGAAAGCAACAGAAGCAAAACAACATCTTCAATGGCTTCGCACCTGAGATCT

CRA1 TACAAGGACGAGAGCAACAGCCACAGAAGAACATTTTCAATGGATTTGGACCCGAGGTTA

: .*. . **:**. ** *** * *. ** ** * . * * .* :

CRC TAGCTCAAGCATTGAAAATTGACGTTCAGTTGGCTCAGCAGCTTCAGAACCAACAAGACA

CRD TCGCGGAAGCATTCAAAATCAACATCGAGACAGCTAAGCAACTACAAAACCAGAAGGACA

CRB TGGCTCAAGCCTTCAAGATCAATGTCGAGACGGCTCAGCAGCTCCAGAACCAGCAAGATA

CRA1 TTGCTCAAGCTTTGAAGATCGATCTTCAGACAGCACAGCAACTTCAGAACCAAGATGACA

* ** **** ** **.** .* * **: .**:.****.** **.*****. * ** *

CRC GCAGAGGAAACATCGTTCGTGTTAAGGGACCTTTCCAGGTCGTGAGGCCACCTCTAAGAC

CRD ACAGAGGAAACATAATCCGAGCAAATGGTCCTCTCCATTTCGTCATCCCACCGCCTCGT-

CRB ACCGTGGCAACATCGTCAAGGTCAACGGACCTTTCGGCGTCATTAGGCCACCCTTGAGAC

CRA1 ACCGTGGAAACATTGTCCGAGTCCAAGGACCGTTCGGTGTCATTAGGCCGCCTTTGAGGG

.*.*:**.***** .* .. * .* **:** ** . **.* * **.** .*

CRC AG-----CCCTACGAGAG----CGAGGAGTGGAGACACCCACGTAGCCCACAGGGCAACG

CRD --GAA-----------------------TGG-------CAGCAAGATGGCATTGCTAATG

CRB GCGG-----------CG----------AAGGCGGCCAACAACCACATGAAATAGCTAATG

CRA1 GCCAGAGACCTCAGGAGGAGGAAGAAGAAGAAGGACGACATG---GACGACACGGTAATG

. *. . ..: * ** *

CRC GCCTTGAGGAGACTATCTGCAGCATGAGGTCCCACGAGAACATTGACGACCCTGCTCGTG

CRD GCATCGAAGAGACTTATTGCACGGCTAAGATTCATGAGAATATCGATGATCCAGAACGGT

CRB GTTTAGAGGAGACTTTGTGCACCATGCGATGCACTGAAAACCTCGATGACCCGTCGGATG

CRA1 GCTTAGAGGAGACCATCTGCAGCGCCAGGTGCACCGATAACCTCGATGACCCGTCTCGTG

* * **.***** :: **** . ...: .. ** ** .* ** ** ** . .

CRC CTGACGTGTACAAGCCCAGCCTAGGTCGCGTGACCAGCGTCAACAGCTATACCTTGCCCA

CRD CTGACCATTTTAGCACACGAGCCGGAAGAATCAGCACTCTTAACAGCCTTAATCTCCCTG

CRB CTGACGTGTACAAGCCATCACTCGGATACATTAGCACACTTAACAGCTACAATCTTCCTA

CRA1 CTGACGTGTACAAGCCACAGCTCGGTTACATCAGCACTCTCAACAGTTACGATCTCCCCA

***** : *: *. .*. .**: ...* * ** * ***** : .. * ** .

CRC TCTTGGAGTATGTCAGGCTCAGTGCCACTCGTGGCGTTCTCCAGGGTAATGCGATGGTGC

CRD TTCTACGTCTAGTCAGACTTAACGCCCTTAGAGGTTATCTCTACAGCGGAGGAATGGTGT

CRB TCCTCAGACTTCTCCGCCTTAGCGCTCTTCGTGGCTCCATCCGTAAAAACGCTATGGTGC

CRA1 TCCTTCGCTTCATCCGTCTCTCAGCCCTCCGTGGATCTATCCGTCAAAACGCAATGGTGC

* * . : **.* ** : ** . .*:** .** . . .. * ******

CRC TTCCTAAATACAACATGAACGCTAACGAGATCTTGTACTGCACTGGAGGACAAGGAAGGA

CRD TGCCACAATGGACGGCAAACGCGCACACGGTGCTATACGTCACAGGAGGTCAAGCCAAGA

CRB TACCGCAATGGAACGTAAACGCAAACGCGGCACTCTACGTGACAAACGGAAAGGCTCATA

CRA1 TTCCACAGTGGAACGCAAACGCGAACGCTATTCTTTACGTGACAGACGGGGAAGCCCAAA

* ** .*.*. *. . .***** .**.. . * *** **:...** *.* .. *

CRC TCCAAGTGGTCAACGACAACGGACAGAACGTGTTGGACCAACAGGTGCAGAAGGGACAGC

CRD TACAAGTGGTGGACGACAATGGTCAGTCGGTGTTCAATGAGCAAGTGGGACAAGGCCAAA

CRB TACAAATGGTGAACGACAACGGAGAAAGAGTGTTCGACCAAGAGATCTCCAGCGGACAGT

CRA1 TCCAGATCGTAAACGACAATGGTAACAGAGTGTTTGACGGACAAGTCTCTCAAGGACAGC

*.**..* ** .******* **: * : ***** .* .. *..* .. **.**.

CRC TCGTGGTCATCCCACAAGGGTTCGCATACGTTGTCCAGTCCCACGGAAACAAGTTCGAGT

CRD TCATTGTGATTCCACAAGGCTTTGCAGTTTCAAAAACGGCTGGTGAAACGGGTTTCGAGT

CRB TACTAGTCGTGCCACAAGGCTTTTCGGTCATGAAACATGCCATAGGCGAACAGTTCGAGT

CRA1 TCATAGCCGTACCACAAGGTTTCTCGGTGGTGAAACGCGCAACAAGCAACCGATTCCAGT

*. * * .* ******** ** *. : .:.. * ..... . *** ***

CRC GGATCTCTTTCAAAACTAATGAAAACGCAATGATCAGCACTTTGGCGGGTAGAACCTCGC

CRD GGATATCATTCAAGACAAACGATAACGCTTACATTAACACACTGAGCGGCCAAACATCGT

CRB GGATCGAATTCAAGACAAACGAAAACGCACAGGTCAACACACTCGCGGGCCGTACCTCAG

CRA1 GGGTTGAGTTCAAAACAAACGCTAACGCGCAAATCAACACTCTGGCGGGACGAACCTCAG

**.* . *****.**:** *.:***** : .* *.***: * . ** ..:**.**.

CRC TCTTGAGGGCATTGCCATTGGAGGTCATATCAAATGGTTTCCAGA---TCTCTCCCGAGG

CRD ACTTGAGAGCAGTTCCAGTGGATGTGAT---CAAAGCGTCATATGGAGTGAACGAGGAAG

CRB TCATGAGAGGTTTGCCGCTTGAGGTTATAACCAATGGGTACCAGA---TCTCTCCCGAAG

CRA1 TCTTGAGAGGTTTACCACTTGAAGTCATAACCAATGGGTTCCAAA---TCTCACCCGAAG

:*:****.* : * **. * ** ** ** .**:* * . * . * :. . **.*

CRC AAGCTAGGAAGATCAAGTTCAACACACTTGAGACCACTTTGACCCGCGCTGCCGGTAGGC

CRD AAGCCAAGAGGATCAAGTTTAGTCAGCAAGAGACCATGTTGTCTATGACACCAAGCTCTT

CRB AAGCTAAACGAGTAAAGTTTAGCACGATTGAGACCACACTGACCC------ATAGCAG--

CRA1 AAGCAAGGAGGGTCAAGTTCAACACGCTCGAGACCACTTTGACTC------ACAGCAGTG

**** *......*.***** *. ....: ******* **:* . . .* :

CRC ----AAC-AACAACAGTTGATCGAGGAGATTGTCGAGGCTTAA

CRD ----------CTTCTTAA-------------------------

CRB -TCCAATGAGCTACGGAAGGCCTAGGGC--TTGA---------

CRA1 GCCCAGCTAGCTACGGAAGGCCAAGGGT--GGCTGCAGCTTAA

*::* ::

The sequences of the primers were the following:

RT Cru FP CTTTACTTGCCCACTTTCTT

RT Cru RP TCCACTTTCTGGTGCATGTC

Similarly, primers for conserved regions of napins SESA1, 2 and 5 were design, as detailed below (yellow boxes):

SESA5 ---ATGGCTAAGCTCATTCTCGTTTTCGCAACTCTTGCCCTCTTCATCCTCCTAGCCAAC

SESA1 ATGGCAAACAAGTTGTTCCTCGTCTGCGCAGCTCTCGCTCTCTGCTTCCTCCTCACCAAC

SESA2 ATGGCAAACAAGCTCTTCCTCGTCTGCGCAACTTTCGCCCTCTGCTTCCTCCTCACCAAC

*** * * ***** * **** ** * ** **** * ******* *****

SESA5 GCATCCATCTACCGCACAGTGGTGGAGTTCGAGGAAGATGATGACGTGAGCAACCCA---

SESA1 GCTTCCATCTACCGCACCGTCGTTGAGTTCGAAGAAGATGACGCCACTAACCCCATAGGC

SESA2 GCTTCCATCTACCGCACTGTTGTCGAGTTCGACGAAGATGACGCCAGCAACCCCATGGGC

** ************** ** ** ******** ******** * * * * *

SESA5 ---CAACAAGGTAAGTGCCAGAGGGAGTTTATGAAGCATCAGCAACTAAGAGGTTGCAAG

SESA1 CCAAAAATGAGGAAATGCCGCAAGGAGTTTCAGAAAGAACAACACCTAAGAGCTTGCCAG

SESA2 CCAAGACA---GAAATGTCAGAAGGAGTTTCAGCAATCACAGCACCTAAGAGCTTGCCAG

* ** ** * * ******* * * ** ** ******* **** **

SESA5 CAATGGATCCGCAAGAGAGCTCAACAAGGAAGAATCGGATACGAAGCTGATGACTTTGAG

SESA1 CAATTGATGCTCCAGCAAGCAAGGCAAGGCCGT------------------AGCGATGAG

SESA2 AAATTGATGCGCATGCAAATGAGGCAAGGCCGTGGTGGTGGTCCCTCCCTCGACGATGAG

*** *** * * * * ***** * * ****

SESA5 CTCACTCTTGATGTCGATCTTGAGGACGATGAGAACCCAATGGGGCCTCAACAACAGTCA

SESA1 TTTGATTTCGAAGACGACATGGAGAACCCACAGGGACAAC------AGCAGGAACAACAG

SESA2 TTCGATTTGGAAGACGACATCGAGAACCCACAAGGCCCCC------AGCAGGGACACCAG

* * * ** * *** * *** ** * * ** ***

SESA5 TCTCTTAAAATGTGTTGTAATGAATTAAGGCAAGTGGACAAGATGTGTGTGTGTCCTACA

SESA1 CTATTCCAGCAGTGCTGCAACGAGCTTCGCCAGGAAGAGCCAGATTGTGTTTGCCCCACC

SESA2 ATCCTCCAGCAGTGCTGCAGCGAGCTTCGCCAGGAAGAGCCAGTTTGTGTTTGCCCCACC

* * *** ** * ** * * ** * ** ***** ** ** **

SESA5 CTGAAAAAGGCGGCTCAACAAGTCAGATTCCAGGGAATGCATGGACAGCAACAGGTGCAA

SESA1 TTGAAACAAGCTGCCAAGGCCGTTAGACTCCAGGGACAGCACCAACCAATGCAAGTCAGG

SESA2 TTGAGACAAGCTGCCAGGGCCGTTAGCCTCCAGGGACAACACGGACCATTCCAATCCAGG

*** * * ** ** ** ** ******** ** ** **

SESA5 CATGTGTTTCAGACTGCTAAGAACTTGCCTAACGTTTGCAAAATCCCTACCGTTGGATCC

SESA1 AAAATTTACCAGACAGCCAAGCACTTGCCCAACGTTTGCGACATCCCGCAAGTTGATGTT

SESA2 AAAATTTACAAGACAGCTAAGTACTTGCCTAACATTTGCAAGATCCAGCAAGTTGGTGAA

* * * **** ** *** ******* *** ***** * **** ****

SESA5 TGCCAGTTCAAGGCATCTCCTTACTAG------------------

SESA1 TGTCCCTTCAACATCCCTTCA------TTCCCTTCTTTCTACTAA

SESA2 TGCCCCTTCCAGACCACCATCCCTTTCTTCCCTCCTTACTAA---

** * *** * *

The sequences of the primers were as follows:

RT napin FP ACCGCTTCCATCTACCGCAC

RT napin RP ATCAATTGCTGGCAAGCTCTTAG
