# Supplementary figures and images for: Transcription Factor DOF4.1 Regulates Seed Longevity in Arabidopsis via Seed Permeability and Modulation of Seed Storage Protein Accumulation
Source: Front Plant Sci. 2022 Jul 1;13:915184. doi: 10.3389/fpls.2022.915184 (PMC9284063; doi:10.3389/fpls.2022.915184)

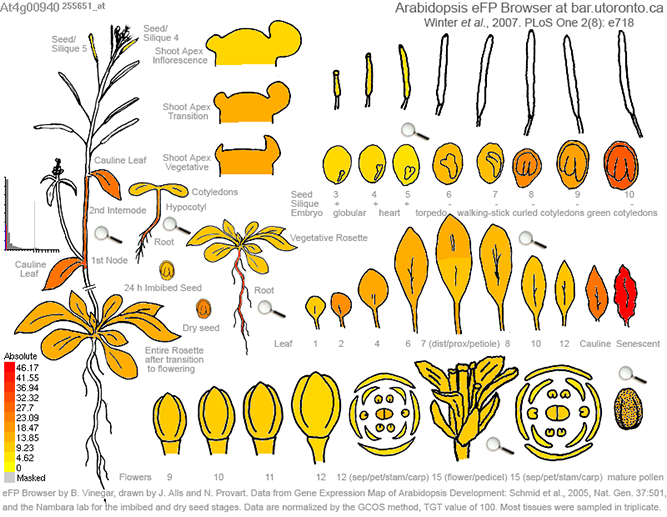

Supplement: Supplementary Figure 1 — Output image from the eFP (electronic fluorescent pictographic) browser showing the expression pattern of DOF4.1 in the Developmental series generated by Schmid et al. (2005, Map of Arabidopsis Development) and colleagues from Detlef Weigel’s group in Tübingen. Each pictograph is colored according to the level of expression for the gene on of interest (Absolute) (http://bar.utoronto.ca/efp2/Arabidopsis/Arabidopsis_eFPBrowser2.html). [file Image_1.TIF]
